# Supplementary material for: CEP128 is involved in spermatogenesis in humans and mice
Source: Nat Commun. 2022 Mar 16;13:1395. doi: 10.1038/s41467-022-29109-7 (PMC8927350; doi:10.1038/s41467-022-29109-7)
Supplement: Supplementary file 1 — Supplementary Information [file 41467_2022_29109_MOESM1_ESM.pdf]

CEP128 is involved in spermatogenesis in humans and mice

Xueguang Zhang *et al*.

Supplementary Information

Supplementary Fig. 1

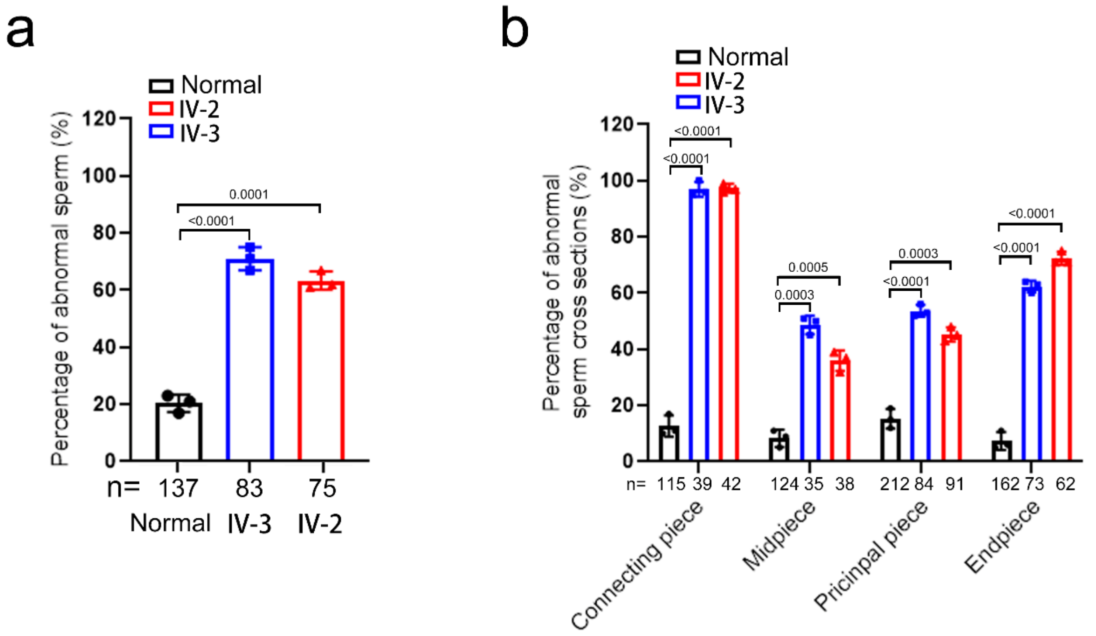

Supplementary Fig. 1 Statistics describing the abnormal phenotype of spermatozoa in the two patients. a Statistics on aberrant sperm in control and patients. Three independent experiments were performed. n, the number of spermatozoa analyzed. (Two-sided student's *t* test; error bars, mean ± SEM). The *p* values are indicated in the graphs. **b** Statistics on abnormal sperm ultrastructure in control and patients. n, the number of cross sections analyzed. Three independent experiments were performed. (Two-sided student's *t* test; error bars, mean ± SEM). The *p* values are indicated in the graphs. Source data are provided as a Source Data file.

Supplementary Fig. 2

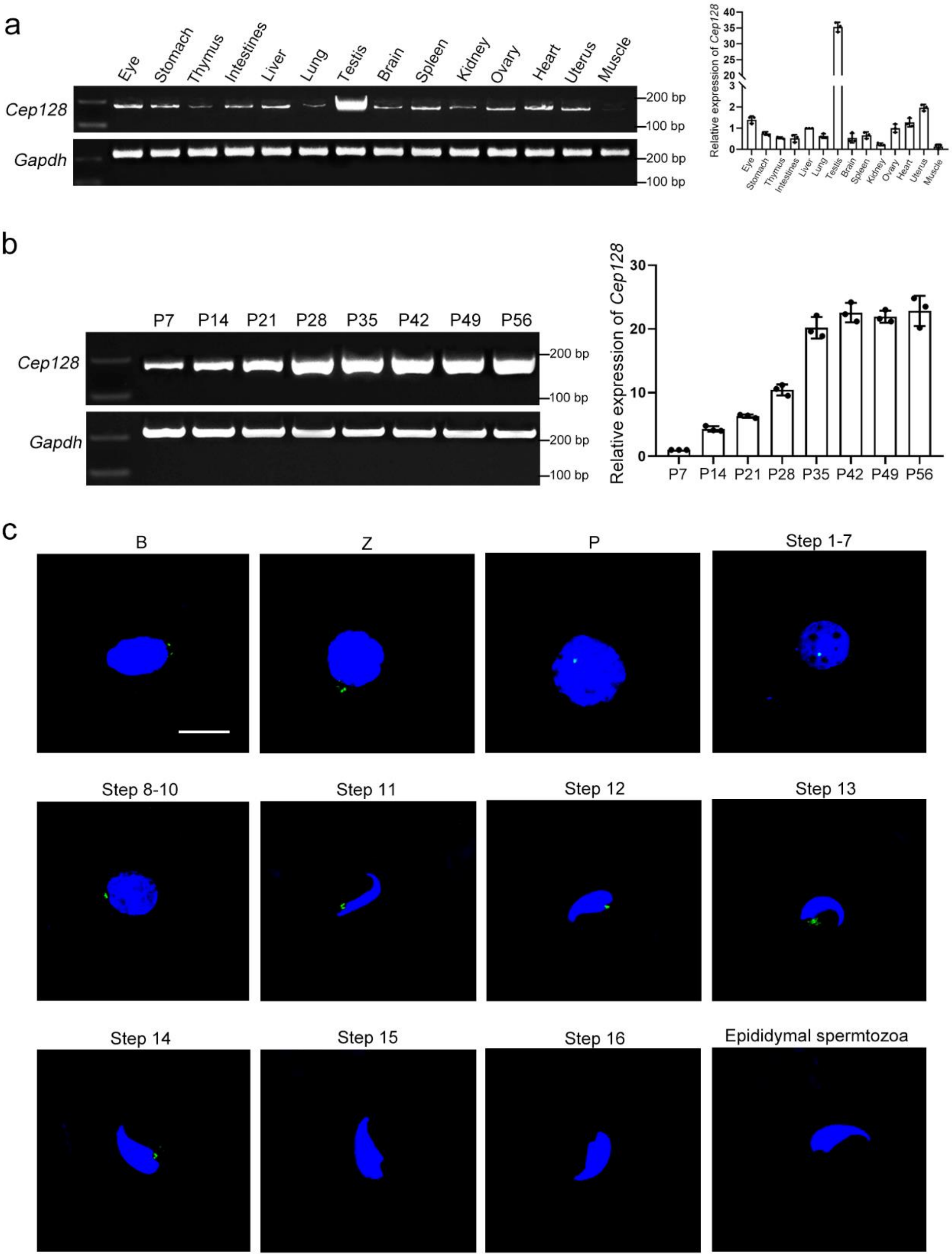

Supplementary Fig. 2 Expression and location of CEP128/*Cep128* in the testis. a qPCR analysis of *Cep128* mRNA expression in various tissues from adult male mice. (n = 3 biologically independent WT mice; error bars, mean ± SEM). **b** *Cep128* expression in mouse testes on various postnatal days. **c** Cep128 was expressed in the

centrioles of B spermatogonia (B), zygotene spermatocytes (Z), pachytene spermatocytes (P), round spermatids (steps 1–7), and elongating/elongated spermatids (steps 8–14); but CEP128 was absent in steps 15–16 spermatids and epididymal spermatozoa. (n = 3 biologically independent WT mice for each age; error bars, mean ± SEM; green, CEP128; blue, DAPI; scale bars, 5 μm). Source data are provided as a Source Data file.

Supplementary Fig. 3

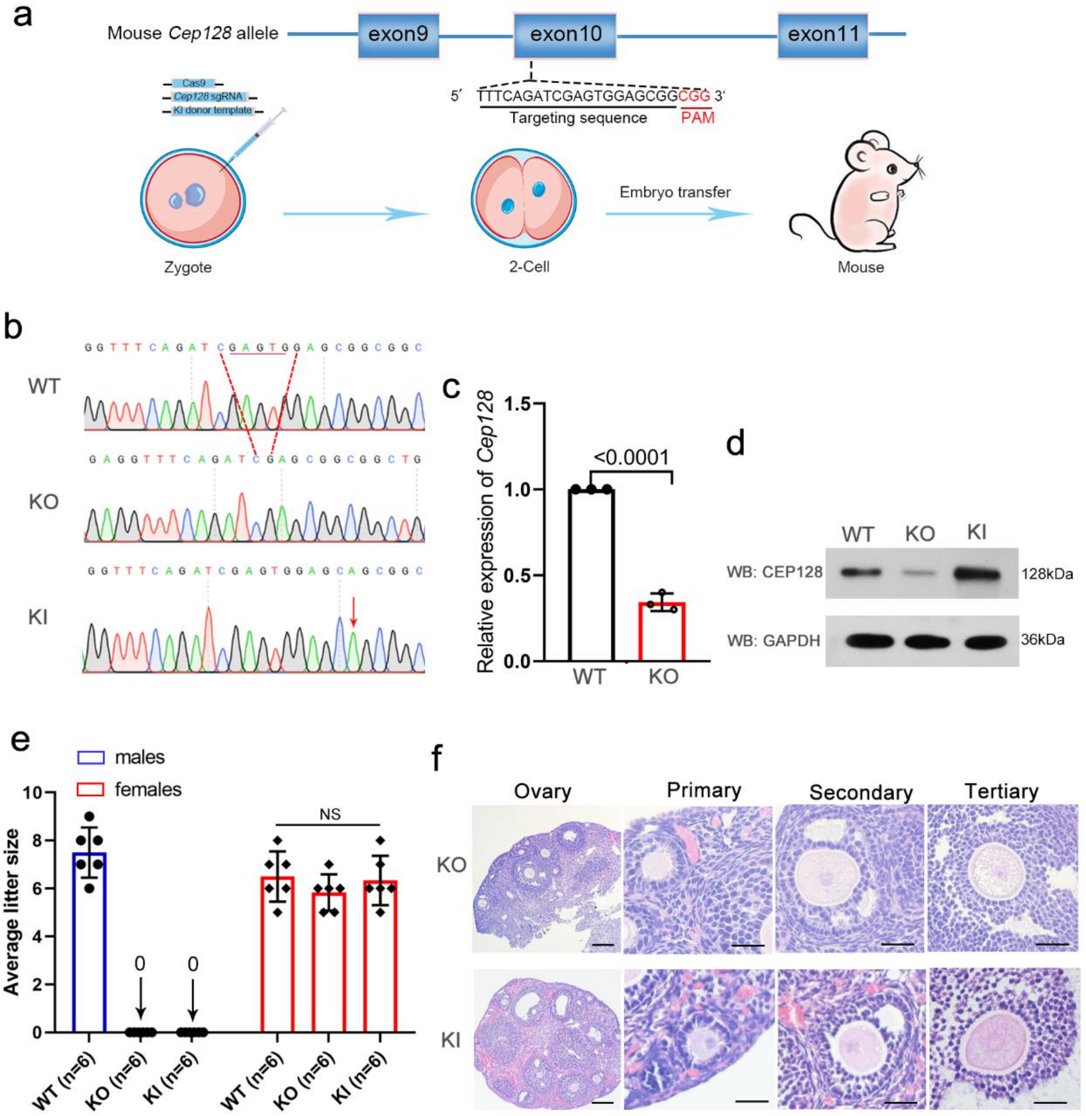

Supplementary Fig. 3 Construction of *Cep128* KO and KI mice. **a** Schematic illustration of the targeting strategy for generating *Cep128* KO and KI mice. **b** Results of PCR-seq genotyping using tail DNA from KO and KI mice. The red line designates the deleted bases in KO mice, and the red arrow denotes the point variant in KI mice. **c** qPCR showed that *Cep128* was significantly reduced in testes from KO mice. (n = 3 biologically independent WT mice or KO mice; two-sided student's t test; error bars, mean ± SEM). The p values are indicated in the graphs. **d** Western blots showing that

CEP128 expression was decreased in the testicular tissues of homozygous KO male mice and increased in homozygous KI mice. (n = 3 biologically independent WT mice, KI mice or KO mice). **e** Fertility in WT, KO, and KI mice. KO and KI males were completely sterile, while females showed normal fertility. n, the number of mice analyzed. (Two-sided student's *t* test; NS, not significant; error bars, mean ± SEM). **f** Dysfunction of *Cep128* exhibited null effects on oocyte development in homozygous KO and KI females (n = 3 biologically independent KO female mice or KI female mice; scale bars, 50 μm). Source data are provided as a Source Data file.

Supplementary Fig. 4

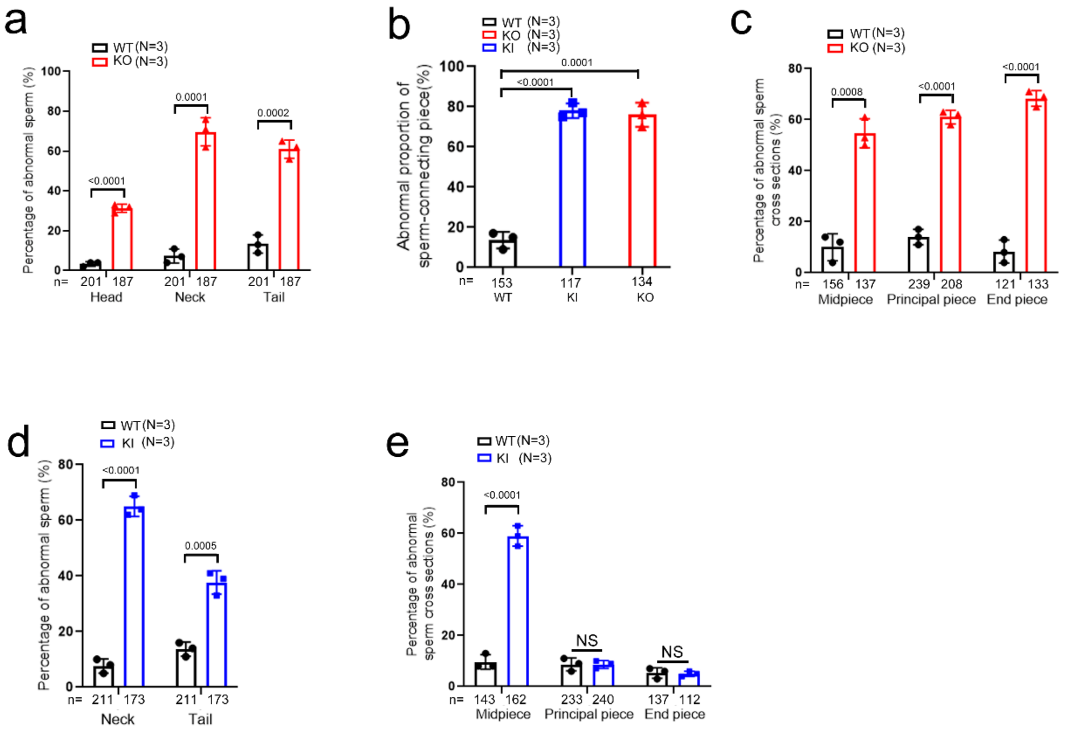

Supplementary Fig. 4 Statistics describing abnormal phenotype of spermatozoa in male homozygous *Cep128* KO and KI mice. a Numbers of morphologically defective spermatozoa in male *Cep128* KO mice. N, the number of mice analyzed. n, the number of sperm analyzed. (Two-sided student's *t* test; error bars, mean ± SEM). The *p* values are indicated in the graphs. **b** Statistics on defects in ultrastructure of the sperm connecting piece in homozygous KO and KI mice. N, the number of mice analyzed. n, the number of cross sections analyzed. (Two-sided student's *t* test; error bars, mean ± SEM). The *p* values are indicated in the graphs. **c** Significant abnormalities detected in sperm flagella of KO mice. N, the number of mice analyzed. n, the number of cross sections analyzed. (Two-sided student's *t* test; error bars, mean ± SEM). The *p* values are indicated in the graphs. **d** Numbers of morphologically defective spermatozoa in

male homozygous KI mice. N, the number of mice analyzed. n, the number of sperm analyzed. (Two-sided student's t test; error bars, mean ± SEM). The p values are indicated in the graphs. **e** Significantly disordered arrangements of MTDs 1–3 and ODFs 1–3 in the midpieces of homozygous KI mice. N, the number of mice analyzed. n, the number of cross sections analyzed. ODF, outer dense fibres; MTD, peripheral microtubule doublets. (Two-sided student's t test; NS, not significant; error bars, mean ± SEM). The p values are indicated in the graphs. Source data are provided as a Source Data file.

Supplementary Fig. 5

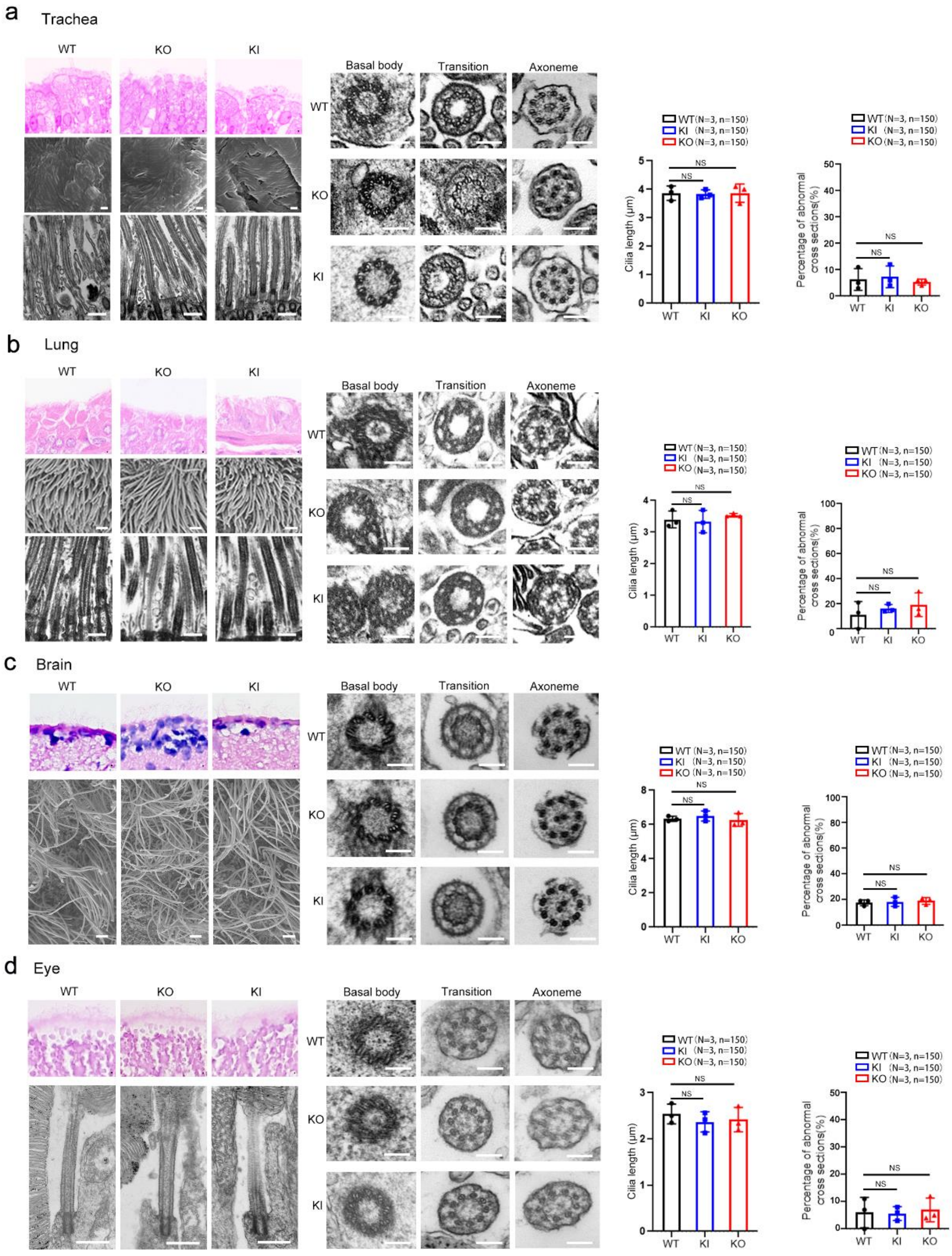

Supplementary Fig. 5 homozygous *Cep128* KO and KI mice show normal ciliary development. a-d Regular development of cilia in trachea (a), lung (b), eye (c), and brain (d) among WT, KO, and KI mice (scale bars, 500 nm/100 nm [TEM]). Data on ciliary length, and abnormal ultrastructure in WT, KO, and KI mice were not

statistically different. N, the number of mice analyzed. n, the number of cilia or cross section analyzed. (Two-sided student's *t* test; NS, not significant; error bars, mean ± SEM). Source data are provided as a Source Data file.

Supplementary Fig. 6

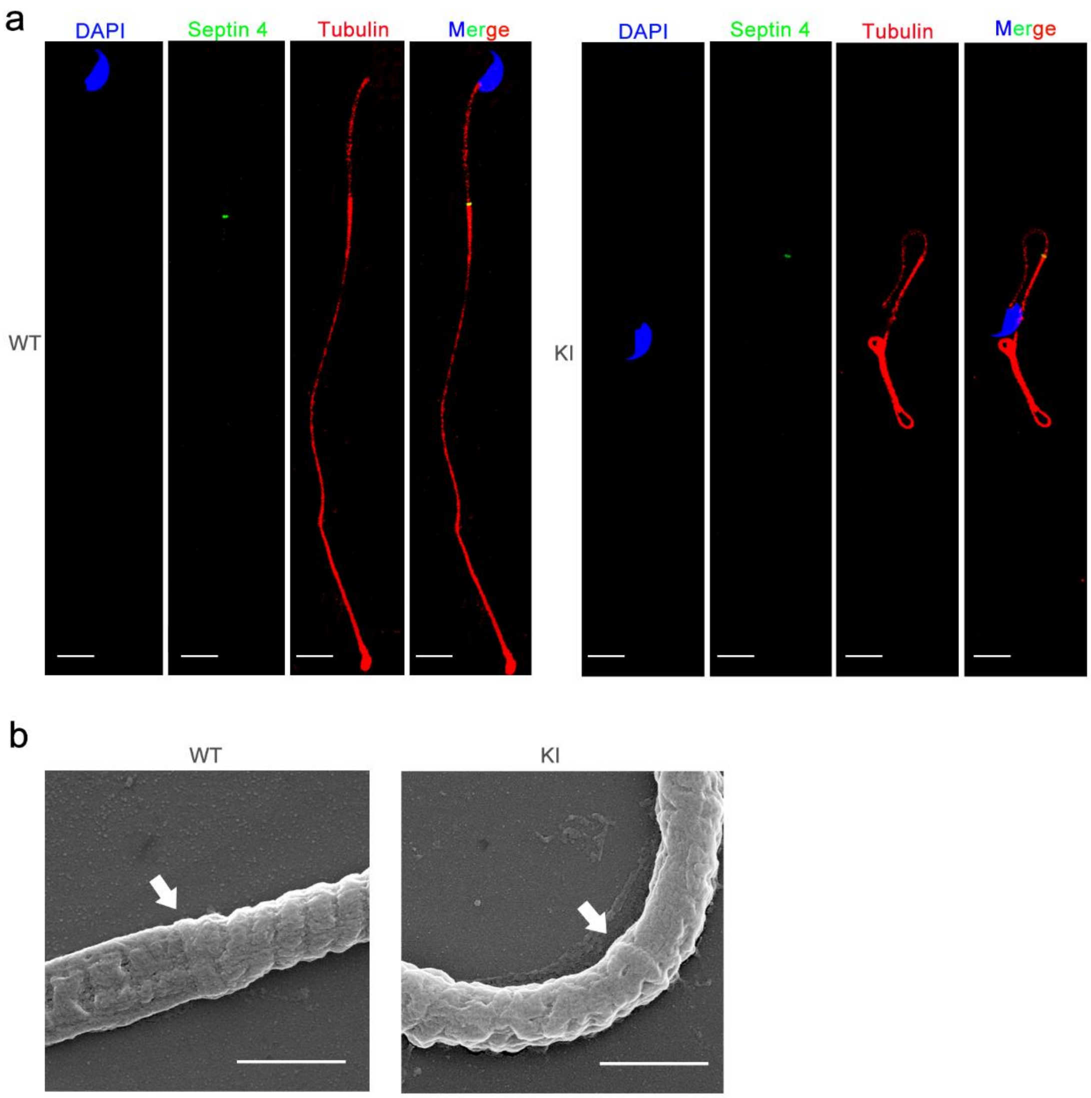

Supplementary Fig. 6 The sperm annulus is normal in male homozygous *Cep128* KI mice. a Expression of Septin 4 was visible in both WT and KI mice (n = 3 biologically independent WT mice or KI mice; green, Septin 4; red, Ac-Tubulin; blue, DAPI; scale bars, 5 μm). **b** SEM showed the intact annulus in sperm from male KI mice (n = 3 biologically independent WT mice or KI mice; scale bars, 1 μm).

Supplementary Fig. 7

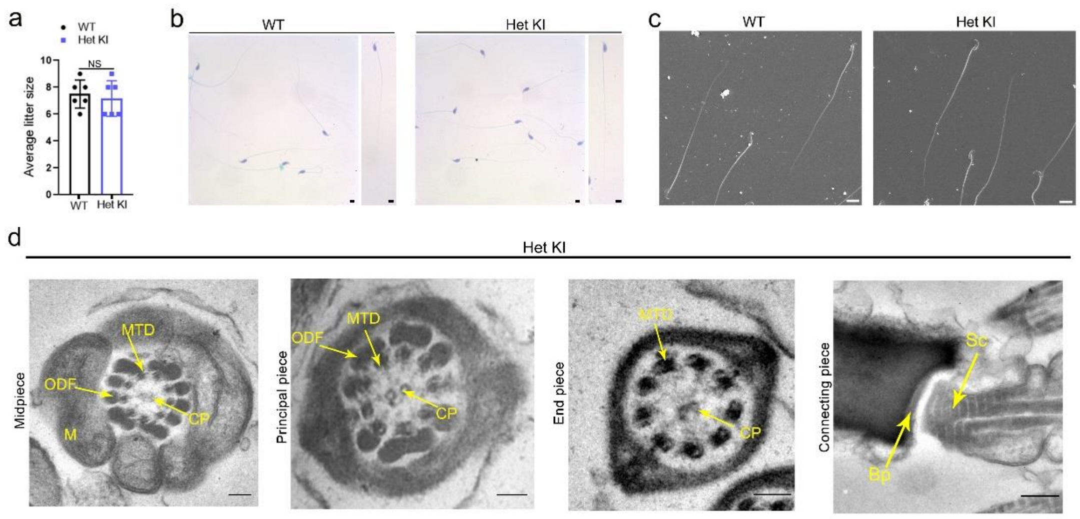

Supplementary Fig. 7. Normal fertility observed in heterozygous KI male mice.

a Heterozygous KI male mice showed normal fertility (n = 6 biologically independent WT mice or heterozygous KI mice; two-sided student's *t* test; NS, not significant; error bars, mean ± SEM). **b** and **c** Papanicolaou staining (b) and SEM (c) showed the normal sperm morphology. (n = 3 biologically independent WT mice or heterozygous KI mice; scale bars, 20 μm). **d** Transmission electron microscopy (TEM) showed the regular "9+2" ultrastructure and sperm-connecting piece of sperm from heterozygous KI mice. Sc, segmented column; Bp, basal plate; CP, central-pair microtubules; ODF, outer dense fibres; MTD, peripheral microtubule doublets; M, mitochondria. (n = 3 biologically independent WT mice or heterozygous KI mice; scale bars, 150 nm). Source data are provided as a Source Data file.

Supplementary Fig. 8

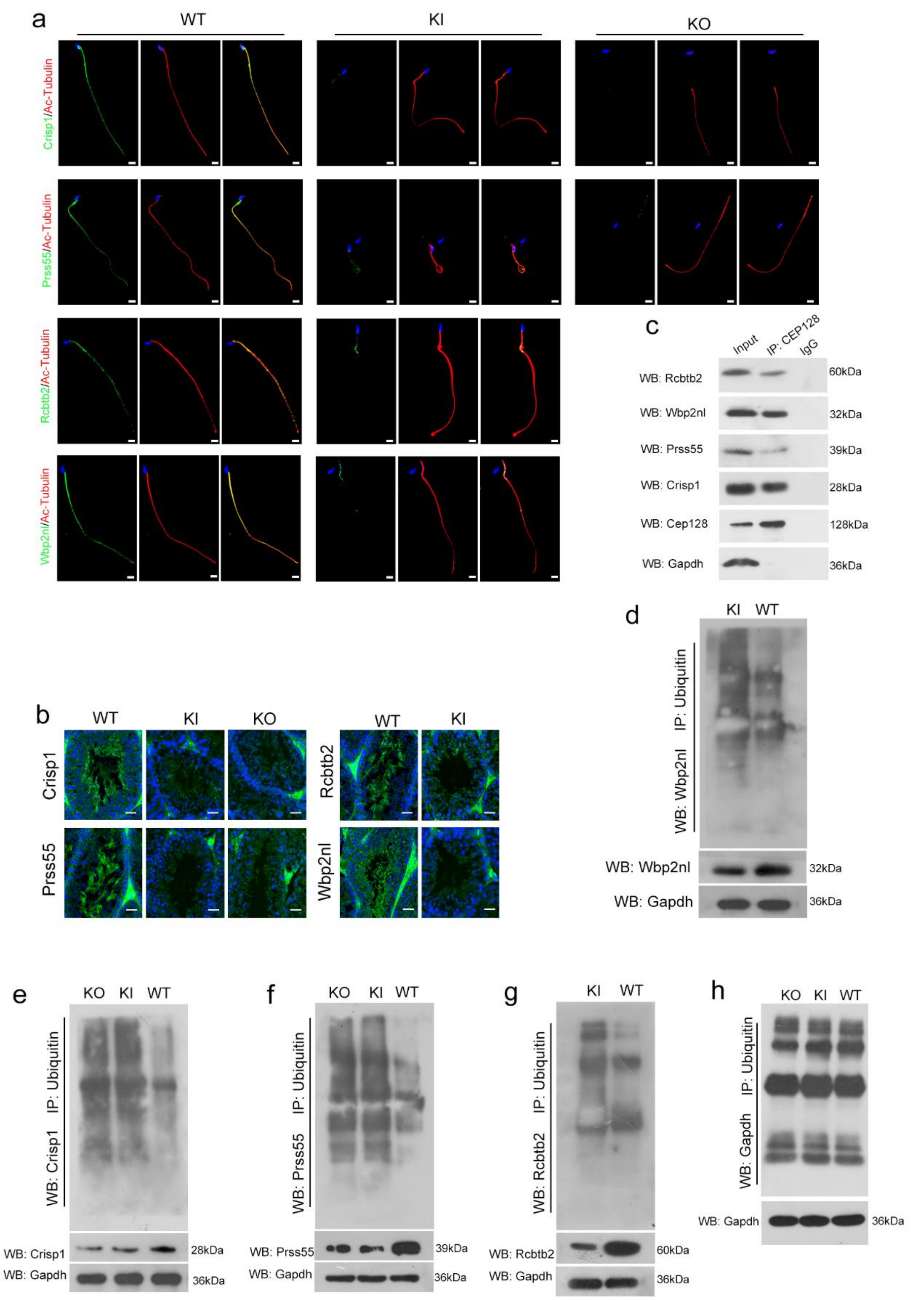

Supplementary Fig. 8 Proteins associated with male fertility are downregulated in male homozygous *Cep128* KO and KI mice. a Staining of Wbp2nl, Rcbtb2, Prss55,

and Crisp1 was marginally detected in the sperm tails from male homozygous KO and/or homozygous KI mice. (n = 3 biologically independent WT mice, KI mice or KO mice; green, Wbp2nl/Rcbtb2/Prss55/Crisp1; red, Ac-Tubulin; blue, DAPI; scale bars, 5 μm). **b** Decreased expression of key proteins in the testes of homozygous KO and/or homozygous KI male mice. (n = 3 biologically independent WT mice, KI mice or KO mice; green, Wbp2nl/Rcbtb2/Prss55/Crisp1; blue, DAPI; scale bars, 20 μm). **c** Binding of CEP128 to Wbp2nl, Rcbtb2, Prss55, and Crisp1 in mouse testes was investigated by co-immunoprecipitation assay. Three independent experiments were performed. **d-h** Increased ubiquitination levels of Wbp2nl (d), Crisp1 (e), Prss55 (f), and Rcbtb2 (g) in homozygous KI and/or KO mice compared with WT mice. No significant difference in ubiquitination levels of Gapdh (h) in homozygous KI and/or KO mice compared with WT mice. The extracted proteins of KO, KI or WT testes were incubated with 3 μg of anti-ubiquitin overnight at 4 °C, anti-RCBTB2 antibody, anti-WBP2NL antibody, anti-CRISP1 antibody, anti-PRSS55 antibody, and anti-GAPDH antibody were used for the immunoblot analysis. Three independent experiments were performed. (n = 3 biologically independent WT mice, KI mice or KO mice). Source data are provided as a Source Data file.

Supplementary Fig. 9

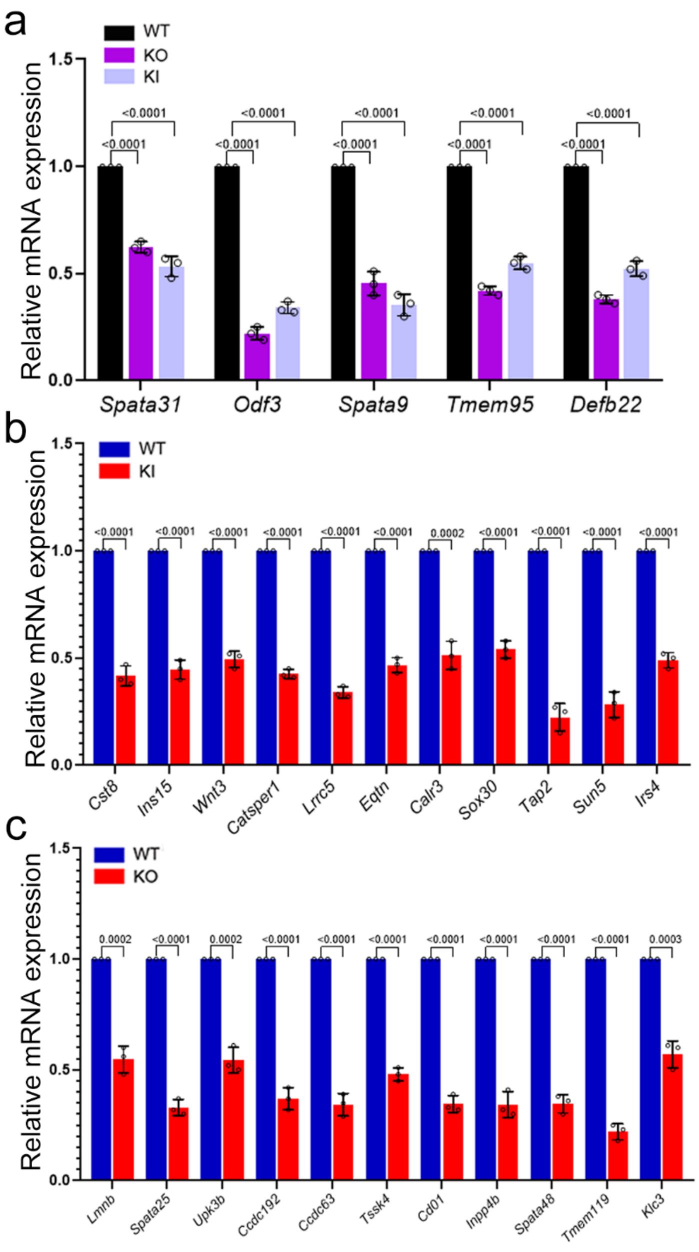

Supplementary Fig. 9 Differentially expressed genes in male homozygous *Cep128* KO and KI mice. a-c Diminution of key genes as determined by RNA-sequencing analysis was further validated by qPCR in both KO and KI mice (a), or only in KI (b) or KO mice (c). (n = 3 biologically independent WT mice, KO mice or KI mice; two-sided student's *t* test; error bars, mean ± SEM). The *p* values are indicated in the graphs. Source data are provided as a Source Data file.

Supplementary Fig. 10

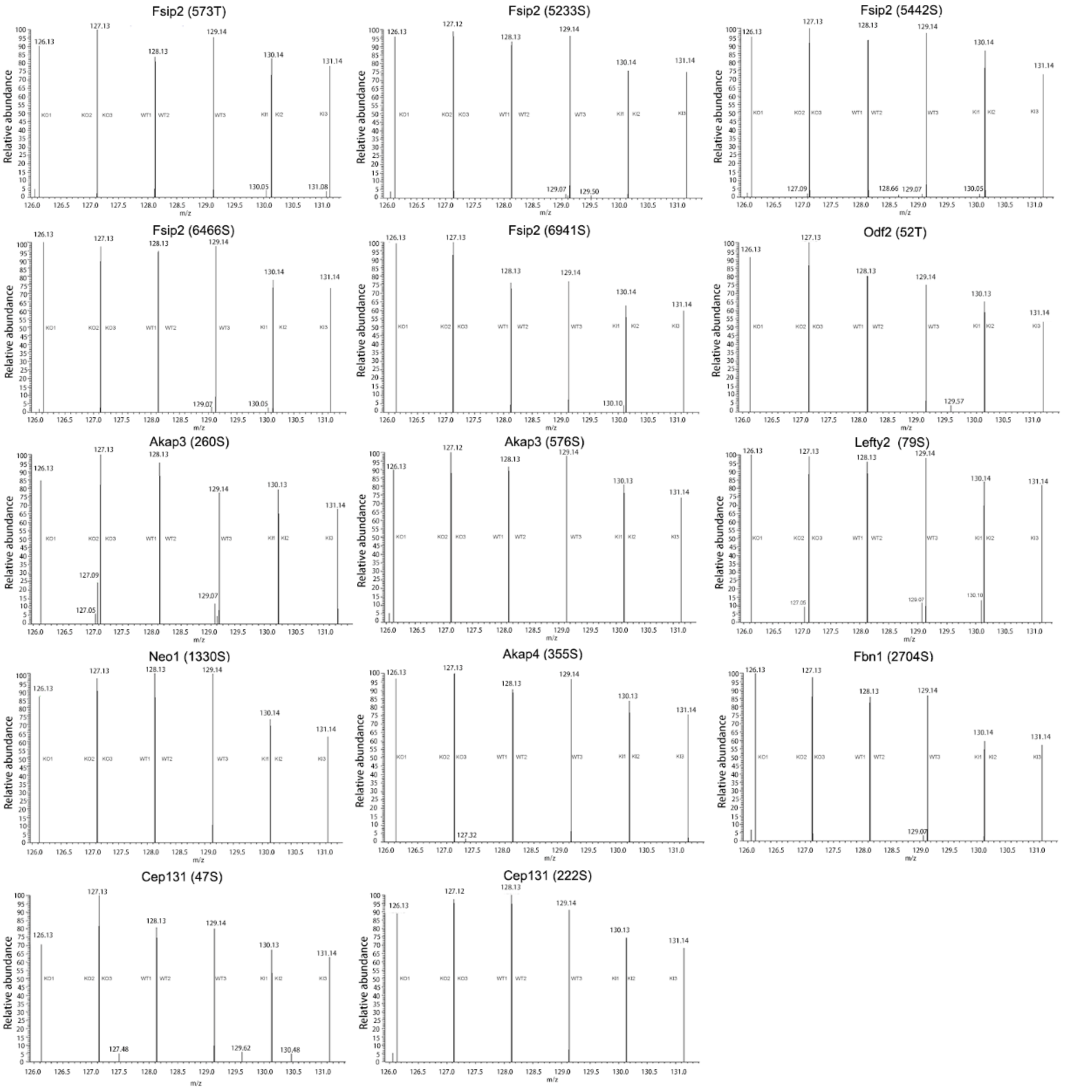

Supplementary Fig. 10 Differential phosphorylated proteins in male homozygous *Cep128* KI mice. MS/MS spectra of differentially phosphorylated proteins involved in spermatogenesis in KI mice compared to WT mice. (n = 3 biologically independent WT mice or KI mice).

Supplementary Fig. 11

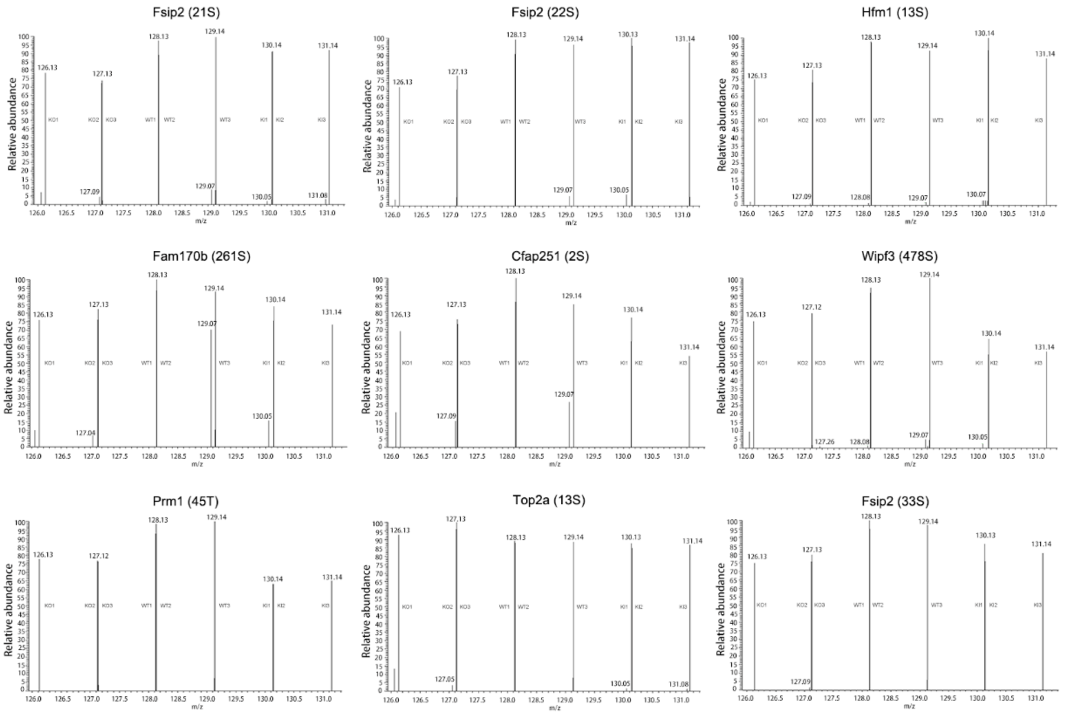

Supplementary Fig. 11 Differential phosphorylated proteins in male *Cep128* KO mice. MS/MS spectra of differentially phosphorylated proteins involved in spermatogenesis in KO mice compared to WT mice. (n = 3 biologically independent WT mice or KO mice).

Supplementary Tables

Supplementary Table 1. The genetic analysis of *CEP128*^{R222Q} variant

	The PAGE Study	geomAD-Exomes	ExAC	1000Genomes	Other projects
Frequency	0.00263 (Global)	0.000689 (Global)	0.000726 (Global)	0.0006 (Global)	8.3KJPN<br>0.02339 (Japanese)
	0.00025 (AfricanAmerican)	0.000 (Europe)	0.000 (Europe)	0.000 (Europe)	
	0.000 (Mexican)	0.00345 (Asian)	0.00346 (Asian)	0.003 (East Asian)	KRGDB<br>0.0113 (Korean)
	0.02 (Asian)	0.00006 (American)	0.00009 (American)	0.000 (American)	
	0.000 (PuertoRican)	0.000 (African)	0.000 (African)	0.000 (African)	A Vietnamese Genetic Variation Database<br>0.008 (Global)
	0.0073 (NativeHawaiian)	0.000 (Ashkenzai Jewish)	0.000 (Other)	0.000 (South Asian)	
	0.000 (Cuban)				
	0.000 (Dominican)				
	0.000 (CentralAmerican)				
	0.000 (SouthAmerican)				
	0.000 (NativeAmerican)				
	0.000 (SouthAsian)				
Function prediction	SIFT<br>Deleterious	PolyPhen-2<br>Possibly Damaging	Mutation Taster<br>Polymorphism	CADD<br>23.3	

NCBI reference sequence number of *CEP128* is GenBank: NM_152446.5. Variants with CADD values greater than 4 are considered to be deleterious.

Supplementary Table 2. Transcription factor binding sites predicted by the JASPAR database.

Model ID	Model name	Score	Relative score	Start	End	Strand	Predicted site sequence
MA0113.1	GR-β	8.78691	0.805386500183	chr14:51187733	chr14:51187750	+	AAGGAGTTTTCGTCCTGT
MA0095.1	YY1	8.38313	0.999999988315	chr9:123835374	chr9:123835379	+	GCCATC
MA0466.1	CEBPβ	8.88441	0.913066988859	chr1:243288486	chr1:243288496	+	CGTTGCTTAAC

Supplementary Table 3. Semen analysis using CASA in the heterozygous KI mice

	Adult Male Mice	
	**WT**	**Het KI**
**Semen parameters**		
Sperm concentration (10^6/ml) [a]	29.83±3.31	37.20±6.51
Motility (%)	53.20±4.67	44.85±6.26
Progressive motility (%)	56.78±9.51	49.50±3.71
**Sperm locomotion parameters**		
Curvilinear velocity (VCL) (μm/s)	60.05±6.11	59.19±4.57
Straight-line velocity (VSL) (μm/s)	19.37±4.51	28.24±4.07
Average path velocity (VAP) (μm/s)	27.56±4.58	36.97±1.11
Amplitude of lateral head displacement (ALH) (μm)	1.01±0.45	0.61±0.33
Linearity (LIN)	0.44±0.12	0.48±0.32
Wobble (WOB, = VAP/VCL)	0.48±0.59	0.47±0.15
Straightness (STR, = VSL/VAP)	0.96±0.55	0.76±0.65
Beat-cross frequency (BCF) (Hz)	7.01±0.37	3.80±0.37

[a] Epididymides and vas deferens

Supplementary Table 4. Clinical features of the couples undergoing intracytoplasmic sperm injection (ICSI) treatment.

Subject		IV-3's wife	IV-2's wife
Age (y)		25-35	25-35
Length of primary infertility history (y)		≥5	≥5
BMI		18-24	18-24
Basal hormones	FSH (IU/L)	2.0	6.1
	LH (IU/L)	0.8	6.9
	E2 (pg/mL)	12.4	61.3
	Prog (ng/ml)	0.66	0.53
Cycle 1	Protocol	Long	Long
	E2 level on the trigger day (pg/mL)	2028	1307
	No. of follicles ≥ 14 mm on the trigger day	5	5
	No. of follicles ≥18 mm on the trigger day	1	4
	No. of oocytes retrieved	8	9
ICSI progress	Oocytes injected	4	7
	Fertilization rate (%)	50% (2/4)	14.3% (1/7)
	Cleavage rate (%)	50% (1/2)	100% (1/1)
	6 cell formation rate (%)	0	0
	8 cell formation rate (%)	0	0
	Blastocyst formation rate (%)	0	0

Supplementary Table 5. Primers used for generation of animal models

Target	Primer sequences （5'—3'）
CAS9-F	TAATACGACTCACTATAGGGAGATTTCAGGTTGGACCGGTG
CAS9-R	GACGTCAGCGTTCGAATTGC
Sg*Cep128*-F	TAATACGACTCACTATAGGGTTTCAGATCGAGTGGAGCGGGTTTTAGAGCTAGAAATAGC
sgRNA-IVT-R	AAAAGCACCGACTCGGTGCCAC
Cep128 donor template oligo	CCCCAGCTGATCATGACGTCTTTCCACCAGCTCTCTTTCTAAGCGCATCTCTCGTTCTATTTCCTGCAGCCGCTGCTCCACTCGATCTGAAACCTCAAGATGAACAGAGAACAAGTAGCATTAGGGAGCTAGCAGGGAGGTGGGGACCGC
Cep128-cha-IF	TAGCTCCCTAATGCTACTTGTTC
Cep128-cha-IR	CAGTTTGCACGGTGTTCTGC

Supplementary Table 6. The qPCR primers used in the present study

Target	Forward primer (5'—3')	Reverse primer (5'—3')	Product(bp)
Cst8	TGGCAGTTGGTGTGGATCAG	TGGTATTCCATTCGGTCTGTG	202
Wnt3	CGCTTCTGCCGCAATTACAT	GTGACTGCGAAGGCGACAC	215
Lrrc52	TATTCCTCTGAACACCCGGAGA	GGAGCTGAGGTCAAGGTAGATG	181
Eqtn	CAGCAACTCTTATTGGGTCCAC	CTCAACGATGAGCTCCTTACC	190
Calr3	CATTGCTCTGGGCCATCTGT	GGTAGTCTGCAGGCCTTTGT	191
Sox30	GAGACTATCCAGATGAGCACACA	CAAGGCTCCAATGTCCAGAGT	140
Tnp2	CCTCAAAGTCACACCAGTAACCA	TTCCTGTTCTTGGGGCAGCTC	197
Sun5	CCGGACGAGGAACATCGGG	GGCAACAGAAGGGGGTCATTC	144
Irs4	GGACAGAGTGACCACAGTGAG	AAGGCTTTGCAGATGACCCTT	169
Catsper1	TGCAGCATTTGCGTGAGTTC	CGGGTCAATACGAGGTCCAC	182
Lmnb2	GCTGCAGAGAACCACATCCA	CAGGGCCAGCTTAATGTCCA	165
Spata25	TTCTTCGGGTTCGTCCCTTG	TTTCTCCGAGGAGTCTCCCA	189
Upk3b	AGACCTGATTGCCTACGTGC	GGTGTCCTTAGTTGAGACATGCT	187
Spata31	TCTGGCAAGAATCTGGGACC	GGATGTTAAATTGTTTTCCATCTCG	219
Ccdc192	TGTTTCGGCTACACTGGCTG	AAGGTGTCTTCGAGCTGAGTG	171
Ccdc63	ACTGAGAAAGACCACGCAGG	TGCTGCAGATTGATGTCCGT	180
Tssk4	TGCTAGGCTTGCCCTACAAC	TAGCAGCTGGAGGATCAGGT	197
Cdo1	CATGATCACACGGACTCCCA	CAACGAAGGAATCAAGACGGT	171
Cep128	GATGCTGAAGGCAGAAAGC	CTCCTTCTTGTCTAGTTCCTCC	178

Inpp4b	AGACGAGCATCAACTGCACA	TGACAAGAAGCAAGGCGACA	167
Spata48	ATCCACCTTCAGTCTTGCCG	GGTTTGGTATGGCTCTGCCT	180
Tmem119	GAAGAAGGCCTGGACACCTC	CCTCTTCCTCCTCCTCCTCC	145
Odf3	ACCAAGTTCAAGGCTCCACAG	TGCCAAAGGTGACAGTAGGA	142
Spata9	CCTGCACAGCGTACAGATGA	TTTGCCAGCACCGCATTAAC	141
Tmem95	CCCCAGATTTCTCGGCCTTT	GTACTGGGGGATCCTGGTCT	155
Insl5	AGACTGTGAAGCTCTGTGGC	CCTGTCTAGGAGCTGGAGGT	140
Klc3	AATCCATCAGGCGAGGAAGC	GTTGAGAGCTGGGAGAGCTG	151
Defb22	CTGGCCCATTTGGTCACAG	CTCGTTGCAGGTTCCGTCT	110
Gapdh	GGTGAAGGTCGGTGTGAACG	CTCGCTCCTGGAAGATGGTG	233
