# Supplementary material for: CEP128 is involved in spermatogenesis in humans and mice
Source: Nat Commun. 2022 Mar 16;13:1395. doi: 10.1038/s41467-022-29109-7 (PMC8927350; doi:10.1038/s41467-022-29109-7)
Supplement: Supplementary file 3 — Description of Additional Supplementary Files [file 41467_2022_29109_MOESM3_ESM.pdf]

Description of Additional Supplementary Files

File name: Supplementary Data 1

Description: The rare clinically relevant variants and rare functionally relevant homozygous variants identified in the proband and his sibling.

File name: Supplementary Data 2

Description: The results of all the proteomic findings of the testes from *Cep128* KO, *Cep128* KI and WT mice in this study.

File name: Supplementary Data 3

Description: The full lists of the differentially regulated genes of the testes from *Cep128* KO, *Cep128* KI and WT mice in this study.

File name: Supplementary Data 4

Description: The differentially phosphorylated sites of the testes from *Cep128* KO, *Cep128* KI and WT mice identified in this study.

File name: Supplementary Movie 1

Description: The WT epididymal sperm were loaded onto a chamber slide for video recording under a phase-contrast microscope. Normal quantity and motility were observed (n = 3 biologically independent WT mice).

File name: Supplementary Movie 2

Description: The homozygous KO epididymal sperm were loaded onto a chamber slide for video recording under a phase-contrast microscope. The sharply reduced motility and increased abnormal morphology were observed in KO sperm (n = 3 biologically independent KO mice).

File name: Supplementary Movie 3

Description: The homozygous KI epididymal sperm were loaded onto a chamber slide for video recording under a phase-contrast microscope. The immotile and defective sperm were observed in KI mice (n = 3 biologically independent KI mice).

File name: Supplementary Movie 4

Description: The heterozygous KI epididymal sperm were loaded onto a chamber slide for video recording under a phase-contrast microscope. The normal sperm movement and count were observed in heterozygous KI mice (n = 3 biologically independent KI mice).
