# Supplementary material for: CEP128 is involved in spermatogenesis in humans and mice
Source: Nat Commun. 2022 Mar 16;13:1395. doi: 10.1038/s41467-022-29109-7 (PMC8927350; doi:10.1038/s41467-022-29109-7)
Supplement: Supplementary file 12 — Reporting Summary [file 41467_2022_29109_MOESM12_ESM.pdf]

Corresponding author(s): Feng Zhang, Xiaohui Jiang, Yihong Yang&Ying Shen

Last updated by author(s): January 26, 2022 NCOMMS-20-44005D

Reporting Summary

Nature Research wishes to improve the reproducibility of the work that we publish. This form provides structure for consistency and transparency in reporting. For further information on Nature Research policies, see our Editorial Policies and the Editorial Policy Checklist.

Statistics

For all statistical analyses, confirm that the following items are present in the figure legend, table legend, main text, or Methods section.

n/a	Confirmed	
☐	☒	The exact sample size (*n*) for each experimental group/condition, given as a discrete number and unit of measurement
☐	☒	A statement on whether measurements were taken from distinct samples or whether the same sample was measured repeatedly
☐	☒	The statistical test(s) used AND whether they are one- or two-sided<br>*Only common tests should be described solely by name; describe more complex techniques in the Methods section.*
☒	☐	A description of all covariates tested
☒	☐	A description of any assumptions or corrections, such as tests of normality and adjustment for multiple comparisons
☐	☒	A full description of the statistical parameters including central tendency (e.g. means) or other basic estimates (e.g. regression coefficient) AND variation (e.g. standard deviation) or associated estimates of uncertainty (e.g. confidence intervals)
☐	☒	For null hypothesis testing, the test statistic (e.g. *F*, *t*, *r*) with confidence intervals, effect sizes, degrees of freedom and *P* value noted<br>*Give P values as exact values whenever suitable.*
☒	☐	For Bayesian analysis, information on the choice of priors and Markov chain Monte Carlo settings
☒	☐	For hierarchical and complex designs, identification of the appropriate level for tests and full reporting of outcomes
☒	☐	Estimates of effect sizes (e.g. Cohen's *d*, Pearson's *r*), indicating how they were calculated

Our web collection on statistics for biologists contains articles on many of the points above.

Software and code

Policy information about availability of computer code

Data collection	*Provide a description of all commercial, open source and custom code used to collect the data in this study, specifying the version used OR state that no software was used.*
Data analysis	Statistical analyses were conducted using GraphPad Prism 8.4.0 software and SPSS 17.0 software. The Burrows Wheeler Aligner (BWA) software and ANNOVAR software were used for WES data analyses.

For manuscripts utilizing custom algorithms or software that are central to the research but not yet described in published literature, software must be made available to editors and reviewers. We strongly encourage code deposition in a community repository (e.g. GitHub). See the Nature Research guidelines for submitting code & software for further information.

Data

Policy information about availability of data

All manuscripts must include a data availability statement. This statement should provide the following information, where applicable:

- Accession codes, unique identifiers, or web links for publicly available datasets
- A list of figures that have associated raw data
- A description of any restrictions on data availability

The proteomic analysis data generated in this study have been deposited in the Proteomics Identifications Database under accession code 'PXD022731' [https://www.ebi.ac.uk/pride/archive/projects/PXD022731]'. The phosphoproteomics data generated in this study have been deposited in the Proteomics Identifications Database under accession code 'PXD025330' [https://www.ebi.ac.uk/pride/archive/projects/PXD025330]. The RNA-seq data generated in this study have been deposited in the SRA Database under accession code 'PRJNA799590' [https://www.ncbi.nlm.nih.gov/Traces/study/?acc=PRJNA799590&o=acc_s%3Aa]'. The whole exon sequencing data generated in this study have been deposited in the SRA Database under accession code 'PRJNA719582' [http://www.ncbi.nlm.nih.gov/bioproject/PRJNA719582].

ExAC Browser (http://exac.broadinstitute.org), 1000 Genomes Project (https://www.internationalgenome.org/), gnomADdatabases (http://gnomad-sg.org/), dbSNP (https://www.ncbi.nlm.nih.gov/snp/), KEGG (https://www.genome.jp/kegg/), Gene Ontology (GO) (http://geneontology.org/), HGMD (http://www.hgmd.cf.ac.uk/ac/all.php).

Field-specific reporting

Please select the one below that is the best fit for your research. If you are not sure, read the appropriate sections before making your selection.

☒ Life sciences ☐ Behavioural & social sciences ☐ Ecological, evolutionary & environmental sciences

For a reference copy of the document with all sections, see nature.com/documents/nr-reporting-summary-flat.pdf

Life sciences study design

All studies must disclose on these points even when the disclosure is negative.

Sample size	The minimal sample size was decided based on our preliminary data we got from the current project to ensure a a statistical power at the level (1-beta) of 80% and a significant level (alpha) of 5% fot t-test.
Data exclusions	No specific data were exluded for the current data set.
Replication	All attempts in this study were repeated three times independently and were successful
Randomization	For experiments other than in vitro culture were randomized, and the no specific method of randomization was used.
Blinding	All the investigators in this study were blinded to group allocation during data collection and/or analysis for experiments

Reporting for specific materials, systems and methods

We require information from authors about some types of materials, experimental systems and methods used in many studies. Here, indicate whether each material, system or method listed is relevant to your study. If you are not sure if a list item applies to your research, read the appropriate section before selecting a response.

Materials & experimental systems

n/a	Involved in the study	
☐	☒	Antibodies
☐	☒	Eukaryotic cell lines
☒	☐	Palaeontology and archaeology
☐	☒	Animals and other organisms
☐	☒	Human research participants
☒	☐	Clinical data
☒	☐	Dual use research of concern

Methods

n/a	Involved in the study	
☒	☐	ChIP-seq
☒	☐	Flow cytometry
☒	☐	MRI-based neuroimaging

Antibodies

Antibodies used

anti-CEP128 (HPA001116, Sigma-Aldrich, WB: 1:1000; IF: 1:200); anti-AcTubulin (ab24610, abcam, IF: 1:1000); anti-NIN (A8215, ABclonal, WB: 1:1000); anti-CEP170 (27325-1-AP, Proteintch, WB: 1:500); anti-YY1 (sc-7341, Santa Cruz Biotechnology, WB: 1:500); anti-GR-β (sc-393232, Santa Cruz Biotechnology, WB: 1:500); anti-SEPT4 (A10238, ABclonal, IF: 1:100); anti-RCBTB2 (13225-1-AP, Proteintech, WB: 1:1000; IF: 1:50); anti-WBP2NL (22587-1-AP, Proteintech, WB: 1:500; IF: 1:50); anti-CRISP1(MAB4675, R&D Systems, WB: 1:500; IF: 1:50); anti-PRSS55 (bs-19443R, Bioss, WB: 1:1000; IF: 1:50); anti-ubiquitin (ab7780, Abcam, WB: 1:500), anti-GAPDH (ab8245, Abcam, WB: 1:1000); anti-CEBPβ (sc-7962, Santa, WB: 1:500); anti-Centriolin (sc-365521, Santa, WB: 1:500). Alexa Fluor 488 (A21206, Thermo Fisher, WB: 1:1000) or Alexa Fluor 594 (A11005, Thermo Fisher, WB: 1:1000). Goat anti-Mouse secondary antibody (Thermo Fisher, G-21040, WB: 1:10000) and Goat anti-Rabbit secondary antibody(Thermo Fisher, 31460, WB: 1:10000).

Validation

The antibodies used in western blotting, Co-IP and immunofluorescence staining are as follows:
1. anti-CEP128 (1:1000, HPA001116, Sigma-Aldrich)
(1) Species specificity: Human
(2) Applications: IHC
(3) Validation: WB analysis in Jurkat and Hs 181 Tes whole cell lysates
(4) References: Lis Jakobsen et al. Novel asymmetrically localizing components of human centrosomes identified by complementary proteomics methods. EMBO J. 2011;30(8):1520-1535.
2. anti-AcTubulin (1:1000, HPA052219, Sigma-Aldrich)
(1) Species specificity: Mouse, Rat, Sheep, Human, Sea urchin
(2) Applications: Flow Cyt, ICC, WB

(3) Validation: Use a concentration of 0.03 - 0.06 µg/ml. Detects a band of approximately 55 kDa (predicted molecular weight: 55 kDa).
3. anti-NIN (1:1000, A8215, ABclonal)
(1) Species specificity: Human, Mouse, Rat
(2) Applications: WB, IF
(3) Validation: Western blot analysis of extracts of Jurkat cells, using NIN antibody (A8215) at 1:1000 dilution. Immunofluorescence analysis of U2OS cells using NIN antibody (A8215) at dilution of 1:100
(4) References: Choi YJ et al. Mutations of ADAMTS9 Cause Nephronophthisis-Related Ciliopathy. Am J Hum Genet. 2019;104(1):45-54.
4. anti-CEP170 (1:500, 27325-1-AP, Proteintch)
(1) Species specificity: Human, Mouse
(2) Applications: WB, IF, ELISA
(3) Validation: HeLa cells were subjected to SDS PAGE followed by western blot with 27325-1-AP (CEP170 Antibody) at dilution of 1:600 incubated at room temperature for 1.5 hours. Immunofluorescent analysis of (-20°C Ethanol) fixed HeLa cells using 27325-1-AP (CEP170 antibody) at dilution of 1:50 and Alexa Fluor 488-Conjugated AffiniPure Goat Anti-Rabbit IgG(H+L).
(4) References: So C et al. A liquid-like spindle domain promotes acentrosomal spindle assembly in mammalian oocytes. Science. 2019;364(6447):eaat9557.
5. anti-YY1 (1:500, sc-7341, Santa Cruz Biotechnology)
(1) Species specificity: Human, Mouse, Rat
(2) Applications: WB, IP, IF, IHC(P), ELISA
(3) Validation: Western blot analysis of YY1 expression in BJAB, MCF7, PC-3, Jurkat, HL-60 and K-562 nuclear extracts. Immunofluorescence staining of formalin-fixed HeLa cells showing nuclear localization. Immunoperoxidase staining of formalin fixed, paraffin-embedded human urinary bladder tissue showing nuclear staining of urothelial cells.
(4) References: Kumaran Satyanarayanan S et al. IFN-β is a macrophage-derived effector cytokine facilitating the resolution of bacterial inflammation. Nat Commun. 2019;10(1):3471.
6. anti-GR-β (1:500, sc-393232, Santa Cruz Biotechnology)
(1) Species specificity: Human, Mouse, Rat
(2) Applications: WB, IP, IF, IHC(P), ELISA
(3) Validation: Western blot analysis of GR expression in HeLa and NIH/3T3 nuclear extracts. Immunofluorescence staining of methanol-fixed HeLa cells showing nuclear and cytoplasmic localization.
(4) References: Hemmer MC et al. E47 modulates hepatic glucocorticoid action. Nat Commun. 2019;10(1):306.
7. anti-Ub (1:500, 10201-2-AP, Proteintech)
(1) Species specificity: Human, Mouse, Rat, Arabidopsis thaliana, Monkey, T. cruzi
(2) Applications: WB, IHC, IF, CoIP, chIP, ELISA
(3) Validation: MCF-7 cells were subjected to SDS PAGE followed by western blot with 10201-2-AP (ubiquitin antibody) at dilution of 1:600 incubated at room temperature for 1.5 hours.
(4) References: Khare S et al. Proteasome inhibition for treatment of leishmaniasis, Chagas disease and sleeping sickness. Nature. 2016;537(7619):229-233.
8. anti-SEPT4 (1:500, A10238, ABclonal)
(1) Species specificity: Human, Mouse, Rat
(2) Applications: WB, IHC, IF
(3) Validation: Western blot analysis of extracts of various cell lines, using SEPT4 antibody (A10238) at 1:1000 dilution.
9. anti-RCBTB2 (1:1000, 13225-1-AP, Proteintech)
(1) Species specificity: Human, Mouse, Rat
(2) Applications: WB, ELISA
(3) Validation: Various lysates were subjected to SDS PAGE followed by western blot with 13225-1-AP (RCBTB2 antibody) at dilution of 1:2000 incubated at room temperature for 1.5 hours.
(4) References: Zhang B et al. Proteomic profiling revealed the functional networks associated with mitotic catastrophe of HepG2 hepatoma cells induced by 6-bromine-5-hydroxy-4-methoxybenzaldehyde. Toxicol Appl Pharmacol. 2011;252(3):307-317.
10. anti-WBP2NL (1:500, 22587-1-AP, Proteintech)
(1) Species specificity: Human, Mouse
(2) Applications: WB, IHC, IF, ELISA
(3) Validation: Human and mouse testis tissue were subjected to SDS PAGE followed by western blot with 22587-1-AP (WBP2NL Antibody) at dilution of 1:600 incubated at room temperature for 1.5 hours.
(4) References: Escoffier J et al. Homozygous mutation of PLCZ1 leads to defective human oocyte activation and infertility that is not rescued by the WW-binding protein PAWP. Hum Mol Genet. 2016;25(5):878-891.
11. anti-CRISP1 (, MAB4675, R&D Systems)
(1) Species specificity: Mouse
(2) Applications: WB, IF
12. anti-PRSS55 (, bs-14058R, Bioss)
(1) Species specificity: Human,
(2) Applications: WB, IF
13. anti-GAPDH (1:1000, ab8245, Abcam)
(1) Species specificity: Mouse, Rat, Rabbit, Human
(2) Applications: WB, ICC/IF
(3) validation: WB analysis in rat and mice
14. anti-CEBPβ (1:500,sc-7962, Santa)
(1) Species specificity: Mouse, Rabbit, Human
(2) Applications: WB, IHC, IP
(3) validation: WB analysis in mice
15. anti-Centriolin (1:500,sc-365521, Santa).
(1) Species specificity: Mouse, Human
(2) Applications: WB, ICC/IF, IP
(3) validation: WB analysis in mice

Eukaryotic cell lines

Policy information about cell lines

Cell line source(s)	hRPE1 cells and HEK 293T cells were obtained from the American Type Culture Collection
Authentication	Cell line authentication were performed by ATCC using COI assay.
Mycoplasma contamination	The cell lines have been tested negative for mycoplasma contamination
Commonly misidentified lines (See ICLAC register)	No cell lines used in this study were found in th database of commonly misidentified cell lines that is maintained by ICLAS and NCBI Biosample.

Animals and other organisms

Policy information about studies involving animals; ARRIVE guidelines recommended for reporting animal research

Laboratory animals	8 weeks C57BL/6 mice (males and females). Mouse housing is in an environment with a room temperature range between 20 and 26 °C under a 12-hour light: 12-hour dark cycle, and a relative ambient humidity at the level of mouse cages of 40%-70%.
Wild animals	The study did not involve wild animals.
Field-collected samples	The study did not involve samples collected from the field.
Ethics oversight	All animal procedures complied with the Animal Care and Use Committee of Sichuan University. The animal experiments were approved by the Experimental Animal Management and Ethics Committee of West China Second University Hospital, Sichuan University.

Note that full information on the approval of the study protocol must also be provided in the manuscript.

Human research participants

Policy information about studies involving human research participants

Population characteristics	1000 healthy men aged 25-35 years old were enrolled as normal controls, who had medical check-up without evidence of any infertility and fathered at least one child.
Recruitment	The brothers with primary infertility and his parents were recruited from West China Second University Hospital, Sichuan University. 1000 healthy men were enrolled as normal controls, who had medical check-up without evidence of any infertility and fathered at least one child. 1000 healthy men were all enrolled from Sichuan, China, and there may be a genetic background selection bias and the frequency of the mutations might be different in various population.
Ethics oversight	All the researches on human subjects obtained ethical approval given by the Ethical Review Board of West China Second University Hospital, and each subject signed informed consent.

Note that full information on the approval of the study protocol must also be provided in the manuscript.
